# Women’s beliefs about medication use during their pregnancy: a UK perspective

**DOI:** 10.1007/s11096-016-0322-5

**Published:** 2016-05-30

**Authors:** M. J. Twigg, A. Lupattelli, H. Nordeng

**Affiliations:** School of Pharmacy, University of East Anglia, Norwich Research Park, Norwich, NR47TJ UK; Pharmacoepidemiology and Drug Safety Research Group, School of Pharmacy, University of Oslo, Oslo, Norway; Division of Mental Health, Norwegian Institute of Public Health, Oslo, Norway

**Keywords:** Beliefs about medicines, Medicines, Pregnancy, Risk perception, United Kingdom

## Abstract

*Background* Previous research has examined the number and extent of medicines taking in pregnant women but not their beliefs and risk perception surrounding their use. *Objective* To describe beliefs and risk perception associated with medicines use for the treatment of common acute conditions among UK women and explore whether this is related to actual medicines use. *Settings* Cross-sectional, web-based study in the UK. *Methods* Pregnant women and mothers within 1 year of giving birth were invited to participate in an online cross-sectional questionnaire-based study via a pregnancy website in the UK. Anonymous data were collected from women regarding their use of medicines (both over-the-counter and prescribed) and their beliefs regarding medicines use during pregnancy. *Main outcome measures* Pregnant women’s beliefs about medicines and their relation to pharmacological treatment of acute conditions in pregnancy. *Results* Pharmacological treatment of conditions in pregnancy ranged from 65.4 % for urinary tract infections (UTIs) to 1.1 % for sleeping problems. Almost three out of ten women avoided using some medications during pregnancy. For heartburn and UTIs, women who did not treat the condition viewed medicines in general as being overused, more harmful and less beneficial, than those who treated the condition. In general, UK pregnant women perceived medicines to be beneficial and slightly overused. *Conclusions* Women’s beliefs about medications impact on treatment of specific conditions in pregnancy such as heartburn and UTIs. Healthcare professionals should explore patient’s beliefs regarding medication at the first maternity care visit to promote appropriate medication use in pregnancy.

**Electronic supplementary material:**

The online version of this article (doi:10.1007/s11096-016-0322-5) contains supplementary material, which is available to authorized users.

## Impact on practice

- Even though the level of treatment varies across various acute pregnancy-related illnesses, many women do not treat them pharmacologically and avoid using medication during pregnancy.
- Women’s risk perception and beliefs about medications seem to be important drivers in the decision-making whether to treat a specific condition during pregnancy with medicines, especially heartburn and UTIs.
- Healthcare professionals should explore patient’s beliefs regarding medication at the first maternity care visit to promote appropriate medication use in pregnancy and hence ensure maternal–fetal health.

## Introduction

Clear guidelines exist for maternity care in the UK [1], however, the current advice regarding medicines use during pregnancy on the National Health Service (NHS) website is to “use as few over-the-counter medicines as possible” [2]. The guidance relating to the use of medicines, issued by the National Institute of Health and Care Excellence (NICE) is vague, suggesting simply that, if necessary, antacids should be used for heartburn and diet and lifestyle changes should be attempted for constipation [1]. Moreover, the package insert leaflets often provide general statements that use in pregnancy should only occur if the benefits outweigh the risks [3]. For both health care professionals and pregnant women themselves, lack of clear recommendations make decisions concerning medication use during pregnancy difficult.

There have been numerous studies investigating the prevalence of medicine use during pregnancy [4–7]. Few studies, however, distinguishing between over-the-counter (OTC) and prescription medicines and report how different conditions in pregnancy are being treated. Currently, the majority of UK research focuses on the use of electronic database to characterise utilisation patterns or to study medication safety in pregnancy in a way that does not explore perceptions of pregnant women regarding taking medication [8–10].

Analysis of international data indicates that women overestimate the risk of taking OTC and prescribed medicines during pregnancy [11]. The authors suggest this may have an impact on whether a woman wants to treat a particular condition during pregnancy. There is a lack of knowledge about the relationship between the woman’s risk perception and her actual medicines use during pregnancy in the UK.

### Aim of the study

The aim of this paper was to describe women’s’ beliefs and risk perception associated with medicines use for certain conditions and whether this relates to actual medication taking. A secondary aim was to describe the extent of medication use according to specific common acute conditions in pregnancy in a UK population and the sources used to obtain information about use during pregnancy.

### Ethical approval

In the UK, ethical approval for the study was received from the University of East Anglia’s Faculty of Medicine and Health Research Ethics Committee dated November 2011 (reference: 2011/2012-02).

## Method

This study is a sub-study of the Multinational Medication Use in Pregnancy Study which has been described in detail elsewhere [12]. Study respondents were recruited via advertisements on national websites commonly used by pregnant women or new mothers in the UK: www.bounty.com (a national charity involved in distributing support information to pregnant women) and Pregnancy Forum www.pregnancyforum.co.uk (a national website for pregnant women) during the period 15th November 2011 to 15th January 2012. The websites were selected on the basis of the number of daily users and subscribers. Women who were interested in participating were directed to the study questionnaire platform by clicking on the study URL in the advertisement. Before the woman could access the on-line questionnaire, she was asked to read the study description. Thereafter, the question “Are you willing to participate in the study?” was posed. Informed consent was given by ticking the answer “yes”. The researchers had no access to these e-mail addresses or personally identifiable data either from the website company or the returned questionnaires. The website company had no knowledge of those women who had completed the questionnaire. Only women who were either pregnant or within 1 year of giving birth were eligible. If they did not meet these criteria they were thanked for their interest and exited from the study.

General information regarding the respondents demographics and activities undertaken during pregnancy e.g. smoking and drinking alcohol were obtained. Health literacy was measured by the Set of Brief Screening Questions (SBSQ) [13] and its use in this study is described in more detail in a previous publication [14]. The SBSQ scale was used as an ordinal variable in the analysis. The SBSQ sum score was dichotomized into low/medium (score 0–9) and high health literacy (score 10–12) due to numbers available for analysis.

Respondents were asked to detail which common conditions they had experienced during pregnancy from a list of common ailments experienced in pregnancy, namely: nausea, heartburn, constipation, common cold, urinary tract infections, pain in the neck or pelvic girdle, headache and sleeping problems. The questions were indication-for-use orientated. The medicines used to treat these common conditions were collected and coded using the World Health Organisation’s ATC indexing system [15]. With the exception of certain common medicines e.g. paracetamol, coding was performed to the fourth level i.e. therapeutic group. Respondents were also asked separately to detail specific uses of OTC medicines and prescription medications during pregnancy and were aided with an OTC example to aid recall.

General beliefs about medicines were obtained using the validated Beliefs about Medicines Questionnaire-General (BMQ-General) [16] with an additional four questions added to reflect the benefits of medicines as described by Horne et al. [17]. Respondents were therefore posed four statements relating to the general overuse of medicines, four relating to the harm associated with taking medicines and four relating to the benefits of medicines. Each item was scored using a 1–5 scale where 1 = strongly disagree and 5 = strongly agree. Responses to each group of four statements were group together to give a score ranging from 4 to 20. Higher scores indicate a higher level of agreement with the constructs represented by each of the sub-scales.

The extent to which respondents would avoid certain medicines was ascertained by asking them to detail if they had deliberately avoided any medicines during their pregnancy and, if so, which ones. The avoided medications were coded using the World Health Organisation’s ATC indexing system [15]. Risk perception was determined by asking respondents to rate their perception of risk of certain common medicines, foods and other substances on the foetus. Respondents were asked to tick a box if they perceived the substances to be not harmful (score = 0) to very harmful (score = 10). A full list can be found in a previous publication [18]. We analysed only the three medication groups that were specifically ask about both regarding use and risk perception; OTC anti-nauseants, paracetamol and antibiotics.

## Data analysis

Descriptive statistics were used to calculate the prevalence of medication use during pregnancy and presented as percentages with 95 % CI. Chi-square tests and Fishers exact tests were performed to investigate the relationship between socio-demographic characteristics and medication use. Where data were normally distributed, means and standard deviations were used along with independent samples *t* tests for comparisons. This was applied when assessing the association between Beliefs about medication (BMQ) and medication use. Where data were not normally distributed or formed from an ordinal scale, medians and interquartiles (IQ) were used along with Mann–Whitney *U* tests for comparisons. This was applied when assessing the association between risk perception and medication use. The significance level was set at *p* < 0.05. The Statistical Package for Social Sciences (SPSS) v21 (IBM SPSS Statistics) was used for the analysis.

In order to assess the sample for generalisability, a comparison was made between the respondent demographics and data available for the national population at the time of the study [19–22].

## Results

### Study population

The UK study sample consisted of 1120 women. The majority (87.8 %) of these were from England with the remaining from Scotland (6.2 %), Wales (4.1 %) and Northern Ireland (1.7 %). Four-hundred and forty-two (39.5 %) were pregnant at the time of completing the questionnaire with the remaining having given birth in the previous year. In those respondents who were not currently pregnant, 34.4 % had a child over 29 weeks old with 46.1 % between 9 and 28 weeks. Of those who smoked before, 205 (72.7 %) gave up once pregnant, 67 (23.8 %) reduced and 10 (3.6 %) either smoked the same or increased. Of the 1120 respondents 193 (17.2 %) reported having a chronic condition of whom 74 (6.6 %) had asthma, 48 (4.3 %) had allergies, 28 (2.5 %) had hypothyroidism, 54 (4.8 %) had depression, 36 (3.2 %) suffered from anxiety and 76 (6.8 %) had another chronic condition. Table 1 shows the demographics of the sample compared to national data for all birthing mothers in the UK at the time of the questionnaire. Women who responded were more likely to be married, have no previous children, drink alcohol and be a non-smokers during pregnancy than the general birthing population in the UK.

**Table 1:** Demographics of the UK sample compared the national birthing population. Adapted from original publication by Lupattelli et al. [12]

Demographic	Study sample % (95 % CI)	General birthing population in the UK^a^
Mean (SD) age	30.5 (5.2)	29.6 [21]
Marital status		
Married	63.3 (60.5–66.1)	53.2 [21]
Parity		
No previous children	48.0 (45.1–50.9)	41.9 [21]
Educational level		
Less than high school	0.6 (0.14–1.05)	16.5 [21]
High School	27.9 (25.3–30.5)	37.2 [21]
More than high school	52.1 (49.2–55.0)	46.3 [21]
Other	19.3 (17.0–21.6)^b^	–
Women smoking before pregnancy	25.2 (22.7–27.7)	19 [22]
Women smoking during pregnancy	7.1 (5.6–8.6)	13.2 [20]
Use of alcohol during pregnancy	28.3 (25.7–30.9)	24.0 [19]

^a^Statistics are for England and Wales only, which account for over 90 % of the study sample

^b^Other education included apprenticeships and other on-the-job training and can mostly be considered as equivalent to the category “Less than high school” and “High school”

### Medication use and treatment of common conditions in pregnancy

In total, 856 women (76.4 %) reported the use of medication (both OTC and prescribed) to treat at least one of the eight common conditions experienced during pregnancy. The most commonly used medications were paracetamol, alginic acid-type drugs e.g. Gaviscon and osmotic laxatives e.g. lactulose. Table 2 illustrates the frequency and treatments used in several common conditions experienced during pregnancy. Although a significant number of respondents experienced these conditions during pregnancy, relatively few of them took medication for nausea (9.5 %), constipation (19.2 %) or sleeping problems (1.1 %). In total, 730 (65.2 %) women reported taking over-the-counter analgesics, used for a variety of conditions including headache, neck pain and pelvic girdle pain. One of the most important aspects of Table 2 concerns the number of women experiencing a UTI [n = 191 (17.1 %)] and not receiving treatment [n = 66 (65.4 %)].

**Table 2:** Common conditions experienced during pregnancy according to medication use

Common condition	N (%) experienced	N (%) treated with conventional medicines	Medicines used to treat condition (with ATC code)	Number (%) reporting prescribed or OTC^a^ use	Number (%) reporting OTC use
Nausea	880 (78.6)	84 (9.5)	Piperazine derivatives e.g. cyclizine (R06AE)	25 (29.8)	14 (16.7)
			Phenothiazines e.g. prochlorperazine (N05AB)	13 (15.4)	0
			Paracetamol (N02BE01)	11 (13.1)	0
Heartburn	832 (74.3)	527 (63.3)	Other drugs for peptic ulcer disease and GORD e.g. alginic acid (Gaviscon) (A02BX)	410 (77.8)	443 (84.1)
			Combinations and complexes of aluminium, calcium and magnesium compounds (A02AD)	137 (26.0)	180 (34.2)
			H2-receptor antagonists, ranitidine (A02BA)	21 (4.0)	18 (3.4)
			Proton pump inhibitors, omeprazole (A02BC)	17 (3.2)	12 (2.3)
			Calcium compounds (A02AC)	16 (3.0)	18 (3.4)
Constipation	619 (55.3)	119 (19.2)	Osmotically acting laxatives e.g. lactulose (A06AD)	71 (60.0)	79 (66.4)
			Bulk-forming laxatives e.g. ispaghula (A06AC)	26 (21.8)	24 (20.2)
			Contact laxatives e.g. senna (A06AB)	22 (18.5)	23 (19.3)
Common cold	622 (55.5)	240 (38.6)	Paracetamol (N02BE01)	214 (89.2)	
Urinary tract infections	191 (17.1)	125 (65.4)	Unspecified antibiotics	76 (60.8)	Not asked
			Penicillins with extended spectrum e.g. amoxicillin (J01CA)	18 (14.4)	Not asked
Pain in neck or pelvic girdle	745 (66.5)	255 (34.2)	Paracetamol (N02BE01)	227 (89.0)	^b^
			Opium alkaloids and derivatives e.g. codeine (N02AA)	10 (3.9)	0
Headache	703 (62.8)	457 (65.0)	Paracetamol (N02BE01)	439 (96.1)	^b^
			Paracetamol/codeine combination product (N02AA)	10 (2.2)	
Sleeping problems	757 (67.6)	8 (1.1)	Paracetamol (N02BE01)	5 (62.5)	Not asked

^a^Respondents were asked to detail the medicines used to treat these common conditions in two sections—the first related to all medicines and could be interpreted as prescribed and over-the-counter (OTC) and the second section specifically asked for OTC medicines. Therefore, there is likely to be some overlap between the two figures

^b^The OTC question asked about all analgesics regardless of condition. This figure is reported separately in the text

A comparison of demographics shows that a greater number of women with higher health literacy, aged under 30 years, married, non-smoking and drinking alcohol during pregnancy treated heartburn with medication when compared to those who did not. Alcohol use was also higher in the group that used medication to treat constipation compared to those who did not. Comparisons for the other conditions and variables listed in the method were not significantly different (E-Table 1).

### Medication avoidance

In total, 815 (72.8 %) women reported deliberately avoiding the use of certain medicines during pregnancy. These were primarily the avoidance of paracetamol [149 (18.3 %)], ibuprofen and combination products [144 (14.7 %)], cough and cold preparations [53 (6.5 %)], antihistamines [36 (4.4 %)] and nasal decongestants [24 (2.9 %)]. The most common reasons for avoiding OTC medicines were: a fear of harming the unborn child [218 women (19.5 %)], medication not recommended [215 (19.2 %)] and stating they would not take any medication during pregnancy/endure as much as possible before taking medication [70 (6.3 %)].

### Beliefs about medicines, risk perception and information needs

Table 3 shows the responses for each of the BMQ-General questions. For the total study population the mean (SD) for the overuse (score of 20 indicates overused), harm (score of 20 indicates harmful) and benefit (score of 20 indicates beneficial) sections were 12.02 (2.69), 9.62 (2.62) and 15.98 (2.23) respectively. Table 4 illustrates the association between beliefs about medicines and medicines used for common conditions. There was a significant difference between women with heartburn and urinary tract infections (UTIs) who took medication and those who did not, in terms of their perception of overuse, harm and benefits associated with medicine taking. Table 4 also compares the risk perception of OTC nausea medication, paracetamol and antibiotics between respondents who took OTC medication for nausea, pain relief and medication for a UTI respectively. In all three groups, women who did not take medication perceived the risk to be greater than those who chose to take medication.

**Table 3:** Pregnant women’s beliefs about medicines in general, n = 1116

Beliefs about medication (BMQ) statement*	% Agree/strongly agree	% Disagree/strongly disagree	% Uncertain
Overuse			
If doctors had more time with patients they would prescribe fewer medicines	43.0	23.0	34.0
Doctors use too many medicines	33.4	28.9	37.8
Natural remedies are safer than medicines	19.6	33.0	47.4
Doctors place too much trust on medicines	24.2	40.0	35.7
Harm			
Most medicines are addictive	17.4	54.3	28.3
People who take medicines should stop their treatment for a while every now and then	22.3	34.9	42.8
Medicines do more harm than good	5.0	67.6	27.5
All medicines are poisons	3.5	77.9	18.6
Benefit			
Without medicines doctors would be less able to cure people	86.4	4.0	9.6
Medicines help many people to live better lives	85.1	2.4	12.4
Medicines help people to live longer	73.0	4.9	22.1
In most cases the benefits of medicines outweigh the risks	69.0	4.2	26.8

* The BMQ general statements as developed by R Horne

**Table 4:** Beliefs about medication (BMQ) and risk perception scores according to specific conditions and medication use

Condition	BMQ section^a^	With condition using medicines	With condition without medicines	*p* value*
		Mean (SD)	Mean (SD)	
Heartburn	Overuse	11.7 (2.6)	12.4 (2.7)	0.001
	Harm	9.3 (2.4)	10.1 (2.5)	<0.001
	Benefit	16.3 (2.1)	15.6 (2.5)	<0.001
Constipation	Overuse	12.0 (2.4)	12.0 (2.6)	0.890
	Harm	9.4 (2.5)	9.6 (2.5)	0.326
	Benefit	16.3 (2.0)	15.9 (2.2)	0.155
Nausea	Overuse	11.7 (2.8)	12.0 (2.7)	0.334
	Harm	9.2 (2.7)	9.6 (2.5)	0.198
	Benefit	16.1 (2.2)	16.0 (2.2)	0.737
UTI	Overuse	11.5 (2.8)	12.6 (2.7)	0.006
	Harm	9.3 (2.7)	10.4 (2.9)	0.014
	Benefit	16.3 (2.2)	14.9 (2.3)	<0.001

Risk perception of^b^	Median (IQ) risk score among women taking medication	Median (IQ) risk score among women not taking medication	*p* value^#^
OTC nausea medication among women with nausea	3 (2–5)	4 (2–5)	0.036
Paracetamol among women having pain conditions	1 (0–2)	2 (1–5)	<0.001
Antibiotics among women with UTI	3 (2–5)	5 (3–7)	0.001

*UTI* urinary tract infection, *OTC* over-the-counter, *IQ* interquartile

* Independent samples *t* test; Overuse, harm and benefit all normally distributed

^#^Data not normally distributed; Mann–Whitney *U* test

^a^Range 4–20 for each section where higher scores indicated higher agreement

^b^Range 0–10 for each medication where higher score indicate perception of greater harm for the foetus

Finally, in terms of obtaining information, 518 (46.3 %) respondents stated they required more information regarding medicines use during their pregnancy. In total, 365 (70.5 %) approached the midwife or nurse for this information with 256 (49.4 %) approaching the physician, 230 (44.4 %) pharmacy personnel and 257 (49.6 %) the internet.

## Discussion

To our knowledge this is the first study specifically exploring the relationship between beliefs and medications use according to specific acute conditions in pregnancy in a UK population with several of our findings important for clinical practice. Firstly we show that a significant number of women are treating common conditions experienced during pregnancy with medication that is either prescribed or purchased over-the-counter. The most concerning figure in this data are the number of women reporting having a UTI and not receiving treatment. Secondly we show that medication use is associated with different socio-demographic factors, particularly in cases of heartburn and constipation. Thirdly, we illustrate the common medicines that are avoided during pregnancy and womens’ reasons for doing so. We go on to show that medicines in general are perceived to be beneficial and slightly overused and that beliefs and risk perception may play an important role in medication use among women with heartburn and UTIs. Finally, pregnant women used a variety of sources when searching for information about medicines, with 50 % choosing to use internet sources.

The disparity in beliefs about medicines and risk perception in respondents indicates a potential lack of awareness of the appropriateness of medicines, particularly in women experiencing heartburn and UTIs and not treating the condition. It is clear that further work should be undertaken to explore these risk perceptions of medicines to understand why women feel these medicines are not safe. This data shows that women viewed medicines as beneficial in general, with fewer suggesting that medicines are overused and harmful when compared to other studies in pregnant women [23].

In those respondents who did not take medicines to treat the UTI, the risk perception aligned well with that from the analysis of the overall international study [11], whereas women treating the condition were significantly below this international average. It may indicate that women need more information regarding the safety of medicines during pregnancy and what is appropriate for them to use, in order to encourage them to treat conditions effectively. Understanding women’s concerns is also essential to promote adherence to prescribed therapy during pregnancy.

The lack of medicines use in women experiencing nausea and constipation is perhaps explained by the relative ease with which these symptoms can be alleviated using diet and lifestyle measures [1, 2]. As this survey did not collect data on severity of conditions or non-medicinal treatments, we cannot evaluate the appropriateness of therapy. However, in those with a UTI, the number not receiving treatment is concerning and potentially links to the respondent’s perception of risk. If left untreated, UTIs can cause significant complications and as such established guidelines exist for the treatment of this acute condition in pregnant women [24].

It is clear from this survey that women have different views regarding their beliefs about medicines and their risk perception of certain medicines for common conditions. This may be having an impact on their willingness to take medicines for these conditions which could lead to a worsening of symptoms or, in the case of untreated UTIs, harm on the fetus. This is particularly visible with the OTC analgesic paracetamol, the use of which during pregnancy is deemed suitable [25]. Yet a large number of women perceived this as a risky medication and would avoid it during their pregnancy. More work needs to be done to explore these concerns with pregnant women at the outset in order to structure a patient-centred consultation around their beliefs and concerns regarding medicines.

Interestingly, the pharmacy is being used as a source of information for medicines equivalent to the number seeking advice from physicians. Currently, pharmacists are not included in the routine care of pregnant women; however, this data shows that they are being used as a source of advice. In previous commentary, it has been stated that pharmacists do not engage in providing information to pregnant women, instead preferring to refer to an appropriate physician, resulting in further delay and appointments for the woman [26]. Therefore, pharmacists should be made aware of these findings and receive adequate professional training in evidence-based treatments in order that they can structure their consultations around the medicines-related beliefs of the patient. There is also, no current guidance readily available for pharmacists and therefore this should be the focus of work in the future to ensure women receive the appropriate treatment at the right time and if necessary are referred to secondary care services. As discussed in the introduction current guidance is vague, with Summaries of Product Characteristics (SPCs) and national formularies often reliant on manufacturer’s warnings rather than evidence behind their use in practice. In the UK, the Teratology Information Service provides guidance on the use of medicines in pregnancy [27] however it is not clear to what extent this is accessed by healthcare professionals.

## Strengths and limitations

This was a large cross-sectional, web-based study conducted in the UK. The most important strength is that we were able to include the women’s perspectives in relation to their medication use in pregnancy. This permits comparison between attitudes and actual medication use during pregnancy. Moreover, the use of an anonymous on-line questionnaire may promote a more truthful answer to sensitive questions [28] and enabled us to reach a large proportion of the birthing population in the UK. Internet penetration rates in the UK are relatively high (85 % with access to internet in their homes and 70 % using the internet every day or almost every day) [29] making this method more applicable compared to countries with lower daily access to the internet. Also, several validated instruments were used including the BMQ and the SBSQ enabling comparison with other prior studies.

However, there are also several limitations that should be acknowledged. Firstly, by using a web-based data collection system we could not calculate a conventional response rate, and the possibility of a self-selection bias cannot be excluded. In order to reduce this risk and reach the widest possible segment of the target population, the invitation to participate in the study was sent to all women subscribed to one website and posted on a pregnancy forum, which were both selected according to the number of daily users/subscribers. Use of pregnancy forums was endorsed given their widespread use among the pregnant population, even of more socially disadvantaged status [30–32]. Recent epidemiological studies indicate reasonable validity of web-based recruitment methods [28, 33]. Hence, the degree to which our findings can be extrapolated to the target population is based on the representativeness of the respondents to the general birthing populations in each country. In most questionnaire-based studies, the study participants are somewhat more resourceful than the general birthing populations. This might also be the case in our study where 7 % of the respondents reported smoking during pregnancy versus 13 % in the general UK birthing population (Table 1). Our findings may therefore be an underestimation of the true occurrence of medication use during pregnancy. Another limitation of this survey is the self-report nature of the responses. The recall of respondents who were towards the latter end of one year post birth may have been a problem in this survey and despite currently being pregnant those women who had not yet given birth would not have been able to provide a full account of their pregnancy. As previously tested for the entire Multinational Medication Use in Pregnancy Study dataset [12], the extent of OTC (8 % difference) and medication use for acute/short-term illnesses (4 % difference) reported by pregnant women was lower than that reported by new mothers. The survey also only asked about medicines-related treatments and did not explore diet and lifestyle modifications that women undertook to treat these common conditions. This would have been useful as most of these common conditions, in the early stages, can be treated with the appropriate changes to diet and lifestyle. Our results should be interpreted with these limitations in mind.

## Conclusion

Beliefs and risk perception play an important role on medication taking patterns in pregnancy. Pregnant women should be encouraged to discuss their concerns about medicines taking with healthcare professionals in order to ensure they receive timely and effective treatment. However, more work needs to be done to determine if healthcare professionals have the tools available to them to allay concerns, assess severity of conditions and provide appropriate treatment advice for pregnant women.

## Electronic supplementary material

Below is the link to the electronic supplementary material.

Supplementary material 1 (DOCX 19 kb)

## References

[1] National Institute for Health and Care Excellence. Antenatal care (CG62) 2008 (updated Feb 2014) [9th Sept 2015]. http://www.nice.org.uk/guidance/cg62/chapter/1-recommendations#management-of-common-symptoms-of-pregnancy.

[2] NHS choices. Medicines in pregnancy 2014 [7th Sept 2015]. http://www.nhs.uk/conditions/pregnancy-and-baby/pages/medicines-in-pregnancy.aspx.

[3] Bjerrum L, Foged A (2003). Patient information leaflets—helpful guidance or a source of confusion?. Pharmacoepidemiol Drug Saf.

[4] Refuerzo JS, Blackwell SC, Sokol RJ, Lajeunesse L, Firchau K, Kruger M (2005). Use of over-the-counter medications and herbal remedies in pregnancy. Am J Perinatol.

[5] Mitchell AA, Gilboa SM, Werler MM, Kelley KE, Louik C, Hernández-Díaz S (2011). Medication use during pregnancy, with particular focus on prescription drugs: 1976–2008. Am J Obstet Gynecol.

[6] Gagne JJ, Maio V, Berghella V, Louis DZ, Gonnella JS (2008). Prescription drug use during pregnancy: a population-based study in Regione Emilia-Romagna, Italy. Eur J Clin Pharmacol.

[7] Twigg M, Holst L, Desborough J, Wright D (2010). Medication-taking behaviour during pregnancy: is it appropriate?. Br J Midwifery.

[8] Charlton R, Snowball J, Sammon C, de Vries C (2014). The clinical practice research datalink for drug safety in pregnancy research: an overview. Thérapie.

[9] Hardy JR, Leaderer BP, Holford TR, Hall GC, Bracken MB (2006). Safety of medications prescribed before and during early pregnancy in a cohort of 81 975 mothers from the UK General Practice Research Database. Pharmacoepidemiol Drug Saf.

[10] Shafe AC, Lee S, Dalrymple JS, Whorwell PJ (2011). The LUCK study: laxative usage in patients with GP-diagnosed Constipation in the UK, within the general population and in pregnancy. An epidemiological study using the General Practice Research Database (GPRD). Ther Adv Gastroenterol.

[11] Petersen I, McCrea RL, Lupattelli A, Nordeng H (2015). Women’s perception of risks of adverse fetal pregnancy outcomes: a large-scale multinational survey. BMJ Open.

[12] Lupattelli A, Spigset O, Twigg MJ, Zagorodnikova K, Mårdby A-C, Moretti ME (2014). Medication use in pregnancy: a cross-sectional, multinational web-based study. BMJ Open.

[13] Chew LD, Bradley KA, Boyko EJ (2004). Brief questions to identify patients with inadequate health literacy. Fam Med.

[14] Lupattelli A, Picinardi M, Einarson A, Nordeng H (2014). Health literacy and its association with perception of teratogenic risks and health behavior during pregnancy. Patient Educ Couns.

[15] WHO. ATC/DDD Index 2015: World Health Organisation; 2015 [23rd Sept 2015]. http://www.whocc.no/atc_ddd_index/.

[16] Horne R, Weinman J, Hankins M (1999). The beliefs about medicines questionnaire: the development and evaluation of a new method for assessing the cognitive representation of medication. Psychol Health.

[17] Horne R, Frost S, Hankins M, Wright S (2001). “In the eye of the beholder”: pharmacy students have more positive perceptions of medicines than students of other disciplines. Int J Pharm Pract.

[18] Lupattelli A, Spigset O, Björnsdóttir I, Hämeen-Anttila K, Mårdby AC, Panchaud A (2015). Patterns and factors associated with low adherence to psychotropic medications during pregnancy—a cross sectional, multinational web-based study. Depress Anxiety.

[19] Sayal K, Heron J, Golding J, Alati R, Smith GD, Gray R (2009). Binge pattern of alcohol consumption during pregnancy and childhood mental health outcomes: longitudinal population-based study. Pediatrics.

[20] The Health and Social Care Information Centre. Statistics on women’s smoking status at the time of delivery: England, Quarter 1 2012/13 2012 [12 Nov 2012]. www.ic.nhs.uk/pubs/wsstd1213q1.

[21] UK National Statistics. Characteristics of Birth 1 and 2/of mother 1 and 2, England and Wales 2010 [6 Nov 2012]. http://www.statistics.gov.uk/hub/population/births-and-fertility/maternities/index.html.

[22] Office for National Statistics. Opinions and lifestyle survey, smoking habits amongst adults, 2012: Office for National Statistics; 2013 [29 April 16]. http://webarchive.nationalarchives.gov.uk/20160105160709/http://www.ons.gov.uk/ons/dcp171776_328041.pdf.

[23] Nordeng H, Koren G, Einarson A (2010). Pregnant women’s beliefs about medications—a study among 866 Norwegian women. Ann Pharmacother.

[24] Hooton T, Gupta K, Calderwood S, Lockwood C, Bloom A. Urinary tract infections and asymptomatic bacteriuria in pregnancy UpToDate2015 [9th Dec 2015]. http://www.uptodate.com/contents/urinary-tract-infections-and-asymptomatic-bacteriuria-in-pregnancy.

[25] Briggs GG, Freeman RK, Yaffe SJ (2005). Drugs used in pregnancy and lactation.

[26] Samuel N, Einarson A (2011). Medication management during pregnancy: role of the pharmacist. Int J Clin Pharm.

[27] UKTIS. BUMPS: Best use of medicines in pregnancy: UK Teratology Information Service; 2016 [30th Mar 2016]. http://www.medicinesinpregnancy.org/.

[28] van Gelder MM, Bretveld RW, Roeleveld N (2010). Web-based questionnaires: the future in epidemiology?. Am J Epidemiol.

[29] Seybert H. Internet use in households and by individuals in 2011. Eurostat Statistics in focus (66/2011) 2011 [29 April 16]. http://ec.europa.eu/eurostat/documents/3433488/5579964/KS-SF-11-066-EN.PDF/090e071f-c3a9-45d8-aa90-9b142251fd3a.

[30] O’Higgins A, Murphy OC, Egan A, Mullaney L, Sheehan S, Turner M (2014). The use of digital media by women using the maternity services in a developed country. Irish Med J.

[31] Bert F, Gualano MR, Brusaferro S, De Vito E, de Waure C, La Torre G (2013). Pregnancy e-health: a multicenter Italian cross-sectional study on internet use and decision-making among pregnant women. J Epidemiol Commun Health.

[32] Kraschnewski JL, Chuang CH, Poole ES, Peyton T, Blubaugh I, Pauli J (2014). Paging “Dr. Google”: does technology fill the gap created by the prenatal care visit structure? Qualitative focus group study with pregnant women. J Med Internet Res.

[33] Ekman A, Dickman P, Klint Å, Weiderpass E, Litton J-E (2006). Feasibility of using web-based questionnaires in large population-based epidemiological studies. Eur J Epidemiol.

