# Supplementary material for: Women’s beliefs about medication use during their pregnancy: a UK perspective
Source: Int J Clin Pharm. 2016 May 30;38:968–76. doi: 10.1007/s11096-016-0322-5 (PMC4929153; doi:10.1007/s11096-016-0322-5)
Supplement: Supplementary file 1 — Supplementary material 1 (DOCX 19 kb) [file 11096_2016_322_MOESM1_ESM.docx]

**E-table 1. Use of the three most commonly reported medication groups by maternal characteristics**

|  | **Used medicines for nausea**  **N= 880 (%)** | | **Medications for heartburn**  **N= 830 (%)** | | **Used medicines for constipation**  **N= 619 (%)** | | **Medications for UTI**  **N= 191 (%)** | |
| --- | --- | --- | --- | --- | --- | --- | --- | --- |
|  | **Yes** | **No** | **Yes** | **No** | **Yes** | **No** | **Yes** | **No** |
| **Health literacy** |  |  |  |  |  |  |  |  |
| *High* | 78.6 | 83.2 | 83.0 | 75.8* | 84.7 | 79.4 | 74.0 | 67.7 |
| *Low/medium* | 21.4 | 16.8 | 17.0 | 24.2 | 15.3 | 20.6 | 26.0 | 32.3 |
| **Age** |  |  |  |  |  |  |  |  |
| *30 years old or less* | 45.2 | 49.7 | 51.6 | 43.0* | 54.6 | 47.6 | 43.2 | 30.3 |
| *31 years old or more* | 54.8 | 50.3 | 48.4 | 57.0 | 45.4 | 52.4 | 56.8 | 69.7 |
| **Marital status** |  |  |  |  |  |  |  |  |
| *Not married* | 3.4 | 6.8 | 5.3 | 10.4* | 4.3 | 7.3 | 12.0 | 6.1 |
| *Married* | 96.6 | 93.2 | 94.7 | 89.6 | 95.7 | 92.7 | 88.0 | 93.9 |
| **Parity** |  |  |  |  |  |  |  |  |
| Previous children | 45.2 | 49.1 | 52.0 | 53.4 | 52.1 | 48.4 | 45.6 | 48.5 |
| *No previous children* | 54.8 | 50.9 | 48.0 | 46.6 | 47.9 | 51.6 | 54.4 | 51.5 |
| **Educational level** |  |  |  |  |  |  |  |  |
| *Up to and including High School* | 25.0 | 28.4 | 30.0 | 30.2 | 30.3 | 28.8 | 36.8 | 37.9 |
| *More than high school* | 54.8 | 52.5 | 52.0 | 48.2 | 52.1 | 54.2 | 48.8 | 37.9 |
| *Others* | 20.2 | 19.1 | 18.0 | 21.6 | 17.6 | 17.0 | 14.4 | 24.2 |
| **Smoking** |  |  |  |  |  |  |  |  |
| *Smoked during pregnancy* | 6.0 | 6.0 | 6.1 | 10.5* | 8.4 | 5.6 | 11.2 | 12.1 |
| *Didn’t smoke during pregnancy* | 94.0 | 94.0 | 93.9 | 89.5 | 91.6 | 94.4 | 88.8 | 87.9 |
| **Alcohol use during pregnancy** |  |  |  |  |  |  |  |  |
| *Used alcohol during pregnancy* | 31.3 | 27.1 | 31.7 | 22.1* | 37.0 | 27.1* | 26.4 | 22.7 |
| *Didn’t use alcohol during pregnancy* | 68.7 | 72.9 | 68.3 | 77.9 | 63.0 | 72.9 | 72.8 | 74.2 |

* Significant values (p<0.05), Fisher’s exact or Chi-square test. Percentages in each box add up to 100.
